# Patient Care Gaps Prior to Maintenance Dialysis Initiation: A Population-Based Retrospective Study

**DOI:** 10.1177/20543581231212134

**Published:** 2023-11-17

**Authors:** Amber O. Molnar, Danielle M. Nash, Jennifer Emblem, Sarah Bota, Eric McArthur, Bin Luo, Yaqing Liu, Amit X. Garg, Peter G. Blake, K. Scott Brimble

**Affiliations:** 1 Division of Nephrology, Department of Medicine, McMaster University, Hamilton, ON, Canada; 2 St. Joseph’s Hospital, Hamilton, ON, Canada; 3 ICES, ON, Canada; 4 Department of Health Research Methods, Evidence, and Impact, McMaster University, Hamilton, ON, Canada; 5 Lawson Health Research Institute, London Health Sciences Centre, ON, Canada; 6 Ontario Renal Network, Ontario Health, Toronto, Canada; 7 Division of Nephrology, Department of Medicine, Western University, London, ON, Canada

**Keywords:** CKD, dialysis, Canadian, clinical nephrology, epidemiology

## Abstract

**Background:**

Guidelines in Ontario, Canada, recommend timely referral for multidisciplinary kidney care to facilitate planned dialysis initiation. Many patients do not receive recommended multidisciplinary kidney care prior to dialysis.

**Objective:**

To better understand why this gap in pre-dialysis care exists, we conducted a study to describe the pathways by which patients initiate maintenance dialysis.

**Design:**

A retrospective cohort study.

**Setting:**

Population-based, using health care administrative databases from Ontario, Canada.

**Patients:**

Adults initiating maintenance dialysis from April 2016 to March 2019.

**Measurements and methods:**

Patients were grouped based on whether they received recommended multidisciplinary kidney care prior to dialysis initiation (at least 1 year of care with at least 2 visits). For those who did not receive recommended care, we grouped patients as having no identified care gap or into the following groups: (1) lack of timely chronic kidney disease (CKD) screening, (2) late nephrology referral (<1 year), or (3) late or no referral for multidisciplinary kidney care among patients followed by a nephrologist for at least 1 year.

**Results:**

A total of 9216 patients were included with a mean (standard deviation) age of 66 (15) years, and 61.5% were male. Of the total, 896 (9.7%) patients died, 7671 (83.2%) remained on dialysis at 90 days, and 649 (7.0%) had stopped dialysis due to kidney function recovery within 90 days. Of the 9216 patients, 5434 (59%) had not received recommended multidisciplinary kidney care. Among those without recommended care, there were 2251 (41.4%) patients with no identified care gaps, 1351 (24.9%) patients with a lack of timely CKD screening, 359 (6.6%) patients with late nephrology referral, and 1473 (27.1%) patients with late or no referral for multidisciplinary kidney care.

**Limitations:**

We could not determine if patients were referred but declined multidisciplinary kidney care.

**Conclusions:**

More than half of patients had not received recommended multidisciplinary kidney care. Many patients experienced an acute decline in kidney function, which may not be preventable, but in others, there were missed opportunities for CKD screening or early referral to nephrology, or at the level of nephrology practice for early referral for multidisciplinary care. This work could be used to inform policies aimed at improving increased uptake of multidisciplinary kidney care prior to dialysis.

## Abrégé

**Contexte:**

Les lignes directrices de l’Ontario (Canada) recommandent que les patients soient dirigés en temps opportun vers une prise en charge multidisciplinaire de la maladie rénale afin de faciliter l’initiation planifiée de la dialyze. Cette recommandation n’est toutefois pas appliquée pour de nombreux patients.

**Objectif:**

Afin de mieux comprendre ce qui explique cette lacune dans les soins de prédialyse, nous avons menu une étude visant à décrire le parcours des patients jusqu’à l’initiation de la dialyse chronique.

**Conception:**

Étude de cohorte rétrospective.

**Cadre:**

Étude populationnelle réalisée à partir des bases de données administratives de santé de l’Ontario (Canada).

**Sujets:**

Adultes ayant amorcé une dialyse d’entretien entre avril 2016 et mars 2019.

**Méthodologie et mesures:**

Les patients ont été groupés selon qu’ils avaient ou non reçu des soins multidisciplinaires recommandés avant l’initiation de la dialyse (au moins un an de soins incluant au moins deux visites). Les patients qui n’avaient pas reçu les soins recommandés ont été groupés soit comme ne présentant aucune lacune de soins identifiée, soit dans l’un des groupes suivants: i) absence de dépistage de l’IRC en temps opportun; ii) référence tardive en néphrologie (<1 an); iii) patients suivis depuis au moins un an par un néphrologue et non référés vers des soins multidisciplinaires ou référés tardivement.

**Résultats:**

En tout, 9 216 patients (61,5 % d’hommes) âgés en moyenne de 66 ans (écart-type: 15 ans) ont été inclus. De cette cohorte, 896 (9,7 %) patients sont décédés, 7 671 (83,2 %) étaient toujours sous dialyse après 90 jours et 649 (7,0 %) avaient cessé la dialyse dans les 90 jours en raison de la récupération de la fonction rénale. Le nombre total de patients n’ayant pas reçu les soins multidisciplinaires recommandés s’élevait à 5 434 (59 %). De ce nombre, 2 251 patients (41,4 %) ont été classés sans lacune de soins identifiée, 1 351 patients (24,9 %) n’avaient pas bénéficié d’un dépistage précoce de l’IRC, 359 patients (6,6 %) avaient été référés tardivement en néphrologie, et 1 473 patients (27,1 %) n’avaient pas été référés en soins multidisciplinaires ou l’avaient été tardivement.

**Limites:**

Nous n’avons pas été en mesure de déterminer si certains patients référés vers des soins multidisciplinaires avaient refusé ces soins.

**Conclusion:**

Plus de la moitié des patients n’ont pas reçu les soins multidisciplinaires recommandés avant la dialyse. De nombreux patients ont vécu une baisse rapide de leur fonction rénale, qui n’aurait possiblement pas pu être évitée, mais dans d’autres cas, des occasions de dépistage précoce de l’IRC ou de référence précoce en néphrologie ont été manquées. Au niveau de la pratique en néphrologie, là encore, des occasions de référence précoce vers des soins multidisciplinaires ont pu être manquées. Ces travaux pourraient éclairer les politiques visant à améliorer la prise en charge multidisciplinaire de la maladie rénale avant la dialyse.

## Introduction

**Figure 1.**
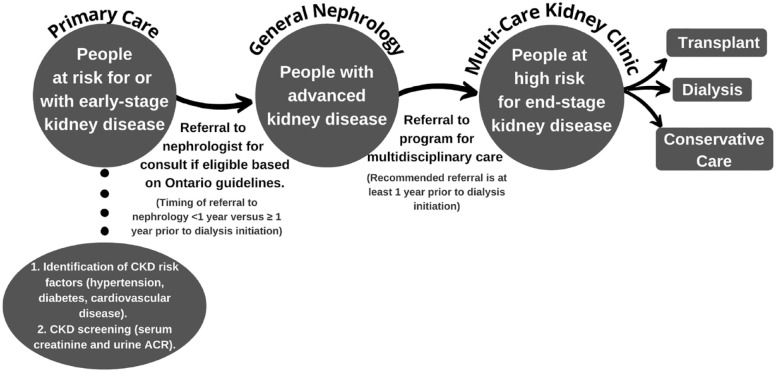
Process of referral to nephrology and for multidisciplinary kidney care. *Note.* CKD = chronic kidney disease; ACR = albumin to creatinine ratio.

Planned dialysis initiation is associated with lower morbidity and mortality.^1-6^ Proper end-stage kidney disease treatment planning requires considerable time to allow for education, shared decision-making, and, if dialysis is selected, creation of a usable access.^7,8^ In Ontario, Canada, these activities usually occur in multidisciplinary kidney clinics, where a team of interdisciplinary health care professionals (eg, nephrologists, nurses, dietitians, pharmacists, socials workers) care for patients with advanced chronic kidney disease (CKD). Receipt of multidisciplinary kidney care is associated with reductions in hospitalizations, unplanned dialysis starts, rate of kidney function decline, and mortality.^9-14^ In Ontario, patients are eligible for multidisciplinary kidney care when their predicted 2-year risk of kidney failure is ≥10%^15^ or estimated glomerular filtration rate (eGFR) is <15 mL/min/1.73 m^2^, and it is recommended that patients receive multidisciplinary kidney care for at least 1 year prior to dialysis initiation.^8^ Nephrologists refer patients to multidisciplinary kidney clinics; therefore, a patient’s primary care physician must first identify CKD and refer the patient to a nephrologist when appropriate (see Figure 1). Prior work by the Ontario Renal Network (ORN) showed that a significant proportion of patients starting maintenance dialysis had not received recommended care in a multidisciplinary kidney clinic.^16^ To better understand why this gap in care exists, we conducted a retrospective study to describe the pathways by which patients initiate maintenance dialysis, with a focus on the pathways and characteristics of patients who did not receive recommended multidisciplinary kidney care. For patients who did not receive recommended care, we grouped patients as having no identified care gap or the following potential care gaps: (1) lack of timely CKD screening, (2) late nephrology referral, and (3) late or no referral for multidisciplinary kidney care among patients receiving care by a nephrologist for at least 1 year.

## Materials and Methods

### Design and Setting

We conducted a descriptive, population-based, retrospective study using administrative health care databases linked using unique encoded identifiers and analyzed at ICES (ices.on.ca/) in Ontario, Canada. The study was conducted according to a pre-specified protocol. The use of data in this project was authorized under section 45 of Ontario’s Personal Health Information Protection Act, which does not require review by a research ethics board. Individual patient consent was not required. The reporting of this study follows the REporting of studies Conducted using Observational Routinely collected health Data (RECORD) guidelines for observational studies (Supplemental Appendix A).^17^

### Data Sources

Patients initiating maintenance dialysis and receiving care in a multidisciplinary kidney clinic in Ontario were identified using the Ontario Renal Reporting System (ORRS) database. Date of dialysis initiation and dialysis dependence at 90 days (as a marker of no kidney function recovery) were confirmed using the Ontario Health Insurance Plan (OHIP) database, which has a sensitivity and positive predictive value for identifying receipt of maintenance dialysis of 100% and 96%, respectively.^18^ Demographics and vital status information were obtained from the Ontario Registered Persons Database. Further details of databases used, including those used to determine comorbidities and physician visits, are outlined in Supplemental Appendix B.

### Study Cohort, Indicators, and Patient Care Groups

**Figure 2.**
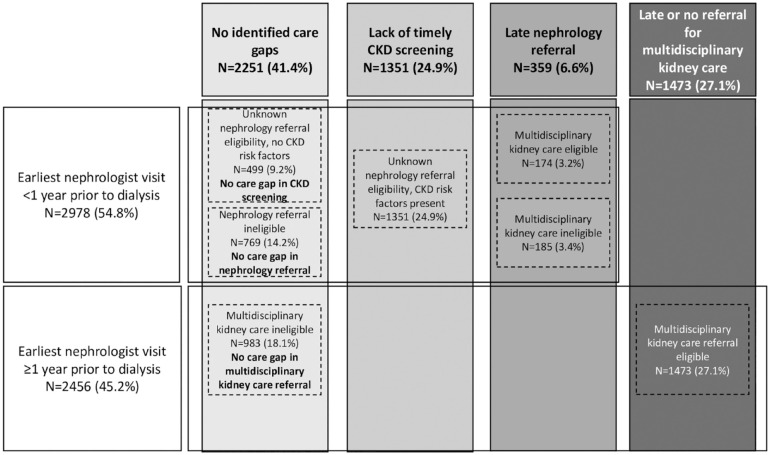
Proportion of patients in each patient care group. *Note.* CKD = chronic kidney disease.

We included adults initiating maintenance dialysis in Ontario, Canada, between April 1, 2016, and March 31, 2019. The date of study inclusion (index date) was the date of dialysis initiation. We excluded patients with a history of maintenance dialysis >90 days prior to the index date, no further evidence of dialysis at >30 days (to exclude those with resolved acute kidney injury [AKI]), and those who received a kidney transplant prior to the index date. As per Ontario CKD care quality metrics, patients were considered to have received recommended multidisciplinary care if their first visit in a multidisciplinary kidney clinic occurred more than 1 year prior to dialysis initiation and if they had 2 or more visits within the past year.^8^ All patients who did not meet these criteria were determined to have not received recommended multidisciplinary kidney care and were grouped using the following indicators: (1) Timing of first nephrologist visit with a look back of 2 years + 90 days prior to dialysis initiation (first visit in <1 year or ≥1year), (2) nephrology referral eligibility in the 1 to 2 years prior to dialysis initiation, based on Ontario-specific criteria (eligible, ineligible, or unknown eligibility),^19^ (3) the presence or absence of CKD risk factors prior to dialysis initiation that should prompt CKD screening according to Ontario recommendations (ie, hypertension, diabetes, cardiovascular disease; see Supplemental Appendix C for definitions),^19^ and (4) multidisciplinary kidney clinic eligibility based on Ontario criteria (eligible or ineligible)^8^ (see Figure 2 and Supplemental Appendix D for further details). These indicators were then used to place patients into the following groups: no identified care gap in CKD screening; lack of timely CKD screening; late nephrology referral; and late or no referral for multidisciplinary kidney care among patients followed by a nephrologist (see Table 1 for further details). Indicator and patient care groups are illustrated in Figure 2.

**Table 1.** Criteria for Patient Care Groups.

Patient care group	Criteria
No identified care gap in CKD screening	Earliest nephrologist visit <1 year prior to dialysis initiation and unknown nephrology referral eligibility ≥1 to 2 years prior to dialysis initiation and CKD risk factors absent >1 year prior to dialysis initiation.
No identified care gap in nephrology referral	Earliest nephrologist visit <1 year prior to dialysis initiation and ineligible for nephrology referral ≥1 to 2 years prior to dialysis initiation.
No identified care gap in multidisciplinary kidney care referral	Earliest nephrologist visit ≥1 year prior to dialysis initiation and were ineligible for multidisciplinary kidney care ≥1 to 2 years prior to dialysis initiation.
Lack of timely CKD screening	Unknown nephrology referral eligibility ≥1 to 2 years prior to dialysis initiation and CKD risk factors were present >1 year prior to dialysis initiation.
Late nephrology referral	First nephrologist visit <1 year prior to dialysis initiation but were eligible for nephrology referral within 1 to 2 years prior to dialysis initiation.
Late or no referral for multidisciplinary kidney care	Earliest nephrologist visit ≥1 year prior to dialysis initiation and eligible for multidisciplinary kidney care ≥1 to 2 years prior to dialysis initiation.

*Note.* CKD = chronic kidney disease.

### Outcomes of Dialysis Dependence and Mortality by Patient Care Groups

We determined the number of patients who remained on dialysis 90 days following dialysis initiation by the presence of ongoing dialysis fee codes (detailed in Supplemental Appendix C) or who died within 90 days of dialysis initiation in each patient care group.

### Descriptive Characteristics Prior to Dialysis Initiation by Patient Care Groups

We compared patient characteristics across patient care groups. We compared demographics, comorbidities (see Supplemental Appendix C for administrative codes), outpatient nephrologist visits and other health care utilization in the past year (ie, primary care and other specialist visits), eGFR in the past 90 and 365 days, rate of eGFR decline in the past 3 years, kidney failure risk at >1 to 2 years, and dialysis initiation characteristics.

### Statistical Analysis

We determined the proportion of patients in each indicator and care group and the proportion of patients in each group who died or remained on dialysis at 90 days. Descriptive characteristics were reported as mean (standard deviation [SD]) and median (interquartile range [IQR]) for continuous variables and as frequency (proportion) for binary and categorical variables and reported by each patient care group. All analyses were conducted using SAS version 9.4 (SAS Institute, Cary, NC).

## Results

There were 10 653 patients who initiated maintenance dialysis in Ontario from April 1, 2016, to March 31, 2019. After exclusions, our final cohort included 9216 patients (Online Supplemental Figure 1). The mean (SD) age was 66 (15) years, and 61.5% were male. There were 896 (9.7%) patients who died, 7671 (83.2%) who remained on dialysis at 90 days, and 649 (7.0%) who had stopped dialysis due to kidney function recovery within 90 days after dialysis initiation. Patients in the no identified care gap in nephrology referral were most likely to die (23.2%) or recover kidney function within 90 days (14.2%) (Online Supplemental Table 1).

Most patients (n = 5434; 59.0%) did not receive recommended multidisciplinary kidney care. Among patients who had not received recommended care, the majority had their earliest nephrologist visit less than 1 year prior to dialysis initiation (n = 2978; 54.8%), among which a minority were eligible for multidisciplinary kidney care (n = 174) in the 1 to 2 years prior to dialysis initiation. Among patients with unknown nephrology referral eligibility due to insufficient serum creatinine or urine albumin to creatinine ratio (ACR) measurements in the 1 to 2 years prior to dialysis initiation, the majority were found to have CKD risk factors (n = 1351). In patients who had not received recommended care but had their earliest nephrologist visit ≥1 year prior to dialysis initiation (n = 2456; 45.2%), more than half were eligible for referral for multidisciplinary kidney care (n = 1473) (Figure 2).

In patients who had not received recommended multidisciplinary kidney care, there were 2251 (41.4%) patients with no identified care gaps (no care gap in CKD screening [9.2%], nephrology referral [14.2%], or multidisciplinary kidney clinic referral [18.1%]). There were 1351 (24.9%) patients with a lack of timely CKD screening, 359 (6.6%) patients with late nephrology referral, and 1473 (27.1%) patients with late or no referral for multidisciplinary kidney care (Figure 2).

Descriptive characteristics compared across patient care groups are presented in Table 2. Patients in the late nephrology referral group were the oldest (mean age 69 years), and patients in the no identified care gap in CKD screening group were the youngest (mean age 52 years). More patients in the no identified care gap in nephrology referral and late nephrology referral groups lived in a rural area (16.8% and 16.2%, respectively). Individuals in the late nephrology referral group had the greatest material deprivation and lived the furthest from dialysis centers.

**Table 2.** Descriptive Characteristics by Patient Care Group.

Characteristic	No identified care gap in CKD screening	No identified care gap in nephrology referral	No identified care gap in multidisciplinary kidney care referral	Lack of timely CKD screening	Late nephrology referral	Late or no referral for multidisciplinary kidney care	Adequate multidisciplinary kidney care
	N = 499	N = 769	N = 983	N = 1351	N = 359	N = 1473	N = 3782
Age, mean (SD)	52 ± 17	67 ± 13	66 ± 15	65 ± 14	69 ± 13	66 ± 14	67 ± 13
Sex (male)	313 (62.7%)	480 (62.4%)	615 (62.6%)	827 (61.2%)	214 (59.6%)	868 (58.9%)	2,353 (62.2%)
Rural (yes)^a^	52 (10.4%)	129 (16.8%)	103 (10.5%)	173 (12.8%)	58 (16.2%)	138 (9.4%)	417 (11.0%)
Material deprivation (ie, proportion of people without a high school diploma, unemployed)^b^							
1 (least deprived)	76 (15.2%)	113 (14.7%)	136 (13.8%)	203 (15.0%)	36 (10.0%)	212 (14.4%)	642 (17.0%)
2	87 (17.4%)	146 (19.0%)	184 (18.7%)	209 (15.5%)	64 (17.8%)	252 (14.4%)	689 (18.2%)
3	99 (19.8%)	152 (19.8%)	172 (17.5%)	257 (19.0%)	66 (18.4%)	252 (17.1%)	675 (17.8%)
4	91 (18.2%)	148 (19.2%)	204 (20.8%)	258 (19.1%)	80 (22.3%)	247 (16.8%)	781 (20.7%)
5 (most deprived)	132 (26.5%)	192 (25.0%)	267 (27.2%)	385 (28.5%)	105 (29.2%)	354 (24.0%)	909 (24.0%)
Missing	14 (2.8%)	18 (2.3%)	20 (2.0%)	39 (2.9%)	8 (2.2%)	22 (1.5%)	86 (2.3%)
Distance to dialysis center from residence (km), median (IQR)^c^	11 (5-30)	12 (5-39)	9 (4-28)	10 (5-31)	14 (5-47)	8 (4-20)	9 (4-22)
Distance to nephrologist from residence (km), median (IQR)^d^	6 (3-18)	8 (4-28)	6 (3-18)	5 (2-19)	6 (2-29)	4 (2-14)	4 (2-13)
Diabetes^e^	0	450 (58.5%)	608 (61.9%)	659 (48.8%)	274 (76.3%)	1,003 (68.1%)	2,334 (61.7%)
Hypertension^e^	0	619 (80.5%)	859 (87.4%)	1,127 (83.4%)	324 (90.3%)	1,336 (90.7%)	3,473 (91.8%)
Cardiovascular disease^e^	0	513 (66.7%)	687 (69.9%)	756 (56.0%)	277 (77.2%)	1,023 (69.5%)	2,565 (67.8%)
AKI^f^	109 (21.8%)	336 (43.7%)	514 (52.3%)	495 (36.6%)	172 (47.9%)	713 (48.4%)	1,276 (33.7%)
CHF^e^	55 (11.0%)	348 (45.3%)	530 (53.9%)	541 (40.0%)	201 (56.0%)	692 (47.0%)	1,443 (38.2%)
Primary care physician visits, mean (SD)^g^	7 ± 11	17 ± 20	15 ± 17	12 ± 14	16 ± 18	13 ± 15	10 ± 12
Outpatient nephrologist visits, mean (SD)^g^	1 ± 2	1 ± 2	3 ± 3	2 ± 2	3 ± 3	5 ± 3	6 ± 2
Cardiologist visits, mean (SD)^g^	1 ± 1	2 ± 4	2 ± 4	1 ± 2	2 ± 4	2 ± 3	2 ± 3
Internal medicine visits, mean (SD)^g^	1 ± 3	2 ± 3	2 ± 3	1 ± 2	2 ± 3	1 ± 3	1 ± 2
Endocrinologist visits, mean (SD)^g^	0.1 ± 0.4	0.3 ± 0.8	0.5 ± 1.1	0.2 ± 0.6	0.4 ± 1.1	0.5 ± 1.1	0.5 ± 1
≥1 multidisciplinary kidney clinic visit^g^	115 (23.0%)	121 (15.7%)	407 (41.4%)	372 (27.5%)	189 (52.6%)	1013 (68.8%)	3782 (100.0%)
eGFR 0-90 days (closest to 90 days) (mL/min/1.73 m^2^), mean (SD)	20 ± 30	26 ± 25	16 ± 14	18 ± 21	12 ± 9	10 ± 6	8 ± 4
Available measurement	323 (64.7%)	683 (88.8%)	827 (84.1%)	992 (73.4%)	335 (93.3%)	1348 (91.5%)	3618 (95.7%)
eGFR 91-365 days (closest to 365 days) (mL/min/1.73 m^2^), mean (SD)	53 ± 38	56 ± 25	32 ± 18	40 ± 28	24 ± 13	18 ± 8	14 ± 5
Available measurement	215 (43.1%)	714 (92.8%)	813 (82.7%)	903 (66.8%)	335 (93.3%)	1393 (94.6%)	3668 (97.0%)
eGFR slope over the past 3 years (ml/min/1.73 m^2^ per year), mean (SD)^h^	−13 ± 2	−14 ± 1	−12 ± 1	−13 ± 1	−12 ± 1	−9 ± 0.2	−6 ± 0.1
Available measurements^h^	66 (13.2%)	754 (98.0%)	796 (81.0%)	624 (46.2%)	350 (97.5%)	1,428 (96.9%)	3,624 (95.8%)
2-year KFRE in the past >1-2 years (closest to 2 years), mean (SD)	—	0.01 ± 0.03	0.03 ± 0.04	0.17 ± 0.23	0.20 ± 0.20	0.30 ± 0.22	0.42 ± 0.22
Available lab data	<6 (<1.2%)	127 (16.5%)	360 (36.6%)	49 (3.6%)	221 (61.6%)	1,242 (84.3%)	3,152 (83.3%)
Dialysis initiation during hospital admission	367 (73.5%)	666 (86.6%)	677 (68.9%)	1,009 (74.7%)	241 (67.1%)	726 (49.3%)	1,232 (32.6%)
Hospitalized at dialysis initiation with ICU visit	171 (34.3%)	324 (42.1%)	271 (27.6%)	435 (32.2%)	97 (27.0%)	223 (15.1%)	343 (9.1%)

*Note.* CKD = chronic kidney disease; SD = standard deviation; IQR = interquartile range; AKI = acute kidney injury; CHF = congestive heart failure; eGFR = estimated glomerular filtration rate; KFRE = kidney failure risk equation; ICU = intensive care unit.

a Defined as communities with ≤10 000 people.

b Material deprivation is associated with poverty and describes access to basic needs. This dimension measures income, quality of housing, education, and family structure characteristics.

c Distance to nephrologist from residence was measured by determining the Euclidean distances (km) between a patient’s postal code and the closest nephrologist’s office.

d Distance to dialysis center from residence was measured by determining the great circle distances (km) between a patient’s postal code and the dialysis facility at the time of dialysis initiation.

e Look-back period was until the beginning of databases (1991).

f Look-back period was 3 years.

g Look back was 1 year.

h At least 2 serum creatinine tests over 3 years were required.

The highest prevalence of diabetes occurred in the late nephrology referral group (76.3%) and was lowest in the lack of timely CKD screening (48.8%). There were no patients with diabetes in the no identified care gap in CKD screening group since diabetes should have prompted screening. Prior AKI was common in all patient care groups, with the highest prevalence in the no identified care gap in multidisciplinary kidney care referral (52.3%) and nephrology referral (43.7%) groups, late nephrology referral (47.9%) group, and late or no referral for multidisciplinary kidney care (48.4%) group. Congestive heart failure (CHF) was most common in the no identified care gap in multidisciplinary kidney care referral (53.9%) and the late nephrology referral (56.0%) groups.

The number of primary care physician visits in the past year was highest for patients in the no identified care gap in nephrology referral and late nephrology referral groups (mean 17 and 16 visits per year, respectively), while patients in the no identified care gap in CKD screening (mean 7 visits per year) and adequate multidisciplinary kidney care (mean 10 visits per year) groups had the lowest numbers. Patients in the no identified care gap in CKD screening or nephrology referral and lack of timely CKD screening groups had the lowest number of nephrologist and other specialist visits. Patients with adequate multidisciplinary kidney care had the highest number of outpatient nephrologist visits in the past year (mean 6 visits per year).

The no identified care gap in CKD screening or nephrology referral groups had higher eGFR prior to dialysis initiation. In the no identified care gap in CKD screening or nephrology referral and lack of timely CKD screening groups, there was a similar, rapid decline in eGFR, as well as a high prevalence of dialysis starts during a hospital admission (73.5%, 86.6%, and 74.7%, respectively) and intensive care unit (ICU) visits during the hospitalization associated with dialysis initiation (34.3%, 42.1%, and 32.2%, respectively).

Compared to patients with adequate multidisciplinary kidney care, those followed up by nephrologists but with late or no referral for multidisciplinary care were more likely to have a history of AKI (48.4% vs 33.7%), a higher rate of eGFR decline (−9 vs −6 mL/min/1.73 m^2^ per year), more dialysis starts during a hospital admission (49.3% vs 32.6%), a higher eGFR in the 90 days prior to dialysis initiation (10 vs 8), and a lower 2-year predicted kidney failure risk (30% vs 42%).

## Discussion

In this retrospective study of 9216 patients who initiated maintenance dialysis, we found that 59% of patients did not receive recommended multidisciplinary kidney care prior to dialysis initiation.

When categorized into patient care groups, we found that almost half of patients who did not receive recommended multidisciplinary kidney care had no identified care gap. Our results support that many of the patients with no identified care gap, especially those followed by a nephrologist for less than a year, experienced a rapid decline in kidney function and then transitioned to chronic dialysis care, which may be difficult to predict and therefore prevent. There was a very high prevalence of dialysis initiation during a hospital admission and during an admission associated with an ICU stay, as well as death, but also kidney function recovery within 90 days after dialysis initiation among those with no identified care gap in nephrology referral; this demonstrates a high level of acute and critical illness in this patient group. Patients in the no identified care gap in CKD screening or multidisciplinary kidney care referral groups were more likely to be dialysis dependent at 90 days and had lower mortality, which may be explained by more rapidly progressive primary kidney disease (eg, glomerulonephritis) and less critical illness resulting in AKI. Due to the acuity and rapid kidney function loss in the no identified care gap groups, it may be very challenging to meaningfully modify their pre-dialysis care.

Patients with a lack of timely CKD screening had a lower prevalence of diabetes and a lower number of health care provider visits; both factors likely created less prompting and opportunity for CKD screening tests to be ordered. This group also had higher material deprivation. Similar to our findings, a recent study from the United Kingdom showed that area-level deprivation is associated with less bloodwork monitoring and outpatient physician visits.^20^ Like the no identified care gap groups, patients with a lack of timely CKD screening experienced a more rapid decline in eGFR and had a high prevalence of inpatient dialysis initiation and dialysis initiation associated with an ICU visit. Therefore, many patients in this group may not have received recommended multidisciplinary kidney care due to an acute illness associated with AKI requiring dialysis. However, education aimed at primary care practitioners regarding how and when to screen for CKD or embedding prompts for CKD screening in electronic medical records could potentially improve care for many patients in this care gap group.^21,22^

A minority of patients were in the late nephrology referral group. This group of patients was slightly older, which has been previously described as a risk factor for late nephrology referral.^23,24^ Surprisingly, these patients had a high number of primary care physician visits, indicating that an overall lack of contact with the health care system was not the main contributor. Patients in this care group had a high prevalence of risk factors for CKD and AKI, such as diabetes and CHF.^25-27^ The very high prevalence of CHF suggests cardiorenal syndrome may be an important contributor to unanticipated dialysis initiation in this patient group.^28,29^ It appears that the kidney failure risk was underappreciated for patients in this group (mean 20% risk in 2 years at 1-2 years prior to dialysis initiation), which delayed referral. Another potential contributor could be the higher material deprivation in this group and their greater distance from nephrology care.^20^ Late referral could be due to patient preference or a tendency for primary care physicians or other specialists to provide CKD care when a nephrologist is harder to access.^30^ Educational strategies aimed at primary care practitioners, along with an electronic consultation practice model and virtual nephrology care, could be of use to improve CKD care in this group.^31-33^

Among patients followed up by a nephrologist for more than a year, 60% were referred late or not at all for multidisciplinary kidney care. This patient group had a high prevalence of prior AKI, which has been previously described as a risk factor for poor prognosis in patients under nephrology care.^34^ The risk of progressing to kidney failure in these patients was likely underestimated by their nephrologist(s), and many likely experienced an unpredictable acute illness leading to AKI requiring dialysis. The underlying reasons why nephrologists did not refer these patients for multidisciplinary kidney care more than a year prior to dialysis despite eligibility could not be determined from the data. Some potential reasons could include individual nephrologist practice patterns and nephrologist clinical judgment independent of eligibility criteria for multidisciplinary kidney care.^8^ Policies that automate or prompt referral for multidisciplinary kidney care in eligible patients could potentially increase uptake of adequate multidisciplinary care for this group.

Prior studies have focused on the timing of nephrology referral (late vs early, based on varying definitions) prior to dialysis initiation.^24^ A recent study using French registry data placed patients into pre-dialysis care trajectories based on health care consumption in the 2 years prior to dialysis initiation. This study also found that patients have heterogenous care pathways prior to dialysis initiation and that late or limited nephrology follow-up and lack of CKD screening are areas to target for improved care, but their analytic approach differed from ours with a primary focus on health care consumption and risk factors for emergency dialysis start.^35^ Our work is unique in that we examined not only timing of nephrologist referral but also multidisciplinary kidney care. The importance of multidisciplinary kidney care for patients approaching dialysis is increasingly recognized.^7^ A kidney failure risk-based approach to the delivery of ambulatory CKD and pre-dialysis care, as was used in this study to group patients by types of potential care gaps, is now being used in multiple jurisdictions.^36,37^ However, our findings highlight the limitations of the kidney failure risk equation, which is best applied to patients with gradually progressive CKD,^38^ and the challenges of implementing high uptake of CKD screening and referral guidelines.

Our study included a large, generalizable, incident dialysis population. There are however some limitations to our study. We could not determine if patients were referred to but declined multidisciplinary kidney care. Similarly, some patients who were referred to nephrology may have declined the consultation or ongoing follow-up. The Ontario Lab Information system, which was used to determine serum creatinine and urine ACR measurements, captures most, but not all, hospitals across the province; therefore, laboratory data from certain hospitals would not be captured, which may have created some misclassification. Similarly, the use of diagnostic codes to determine CKD risk factors may have resulted in some misclassification.

In conclusion, more than half of the patients in our cohort had not received recommended multidisciplinary kidney care prior to dialysis initiation, and almost half of these patients had no identified care gap. Future work could focus on tools to better identify patients at risk of dialysis who are not well captured by current kidney failure risk-prediction tools.^38^ For example, a history of AKI could be a very important risk factor in certain populations, as prior AKI was highly prevalent in our cohort. One might consider adding a history of AKI or CHF as risk factors that prompt screening for CKD and/or earlier referral to nephrology and multidisciplinary kidney clinics. Future work could also focus on educational and collaborative strategies with primary care to optimize CKD screening and nephrology referral and policies that automate or prompt referral into multidisciplinary kidney care for patients followed by nephrologists.

## Supplemental Material

sj-docx-1-cjk-10.1177_20543581231212134 – Supplemental material for Patient Care Gaps Prior to Maintenance Dialysis Initiation: A Population-Based Retrospective Study Click here for additional data file. Supplemental material, sj-docx-1-cjk-10.1177_20543581231212134 for Patient Care Gaps Prior to Maintenance Dialysis Initiation: A Population-Based Retrospective Study by Amber O. Molnar, Danielle M. Nash, Jennifer Emblem, Sarah Bota, Eric McArthur, Bin Luo, Yaqing Liu, Amit X. Garg, Peter G. Blake and K. Scott Brimble in Canadian Journal of Kidney Health and Disease
