# Supplementary material for: Patient Care Gaps Prior to Maintenance Dialysis Initiation: A Population-Based Retrospective Study
Source: Can J Kidney Health Dis. 2023 Nov 17;10:20543581231212134. doi: 10.1177/20543581231212134 (PMC10657522; doi:10.1177/20543581231212134)
Supplement: sj-docx-1-cjk-10.1177_20543581231212134 – Supplemental material for Patient Care Gaps Prior to Maintenance Dialysis Initiation: A Population-Based Retrospective Study [file sj-docx-1-cjk-10.1177_20543581231212134.docx]

**Supplemental Material**

Table of Contents

**Supplemental Table 1: Dialysis dependence and mortality by patient care groups**

**Supplemental Figure 1: Cohort selection**

**Appendix A: RECORD reporting**

**Appendix B: Databases**

**Appendix C: Diagnostic and procedural codes**

**Appendix D: Indicator definitions**

**Supplemental Table 1: Dialysis dependence and mortality by patient care groups**

| **Outcome** | **Total** | **No identified care gap in CKD screening** | **No identified care gap in nephrology referral** | **No identified care gap in multidisciplinary kidney care referral** | **Lack of timely CKD screening** | **Late nephrology referral** | **Late or no referral for multidisciplinary kidney care** | **Adequate multidisciplinary kidney care** |
| --- | --- | --- | --- | --- | --- | --- | --- | --- |
| All-cause mortality | 896 (9.7%) | 54 (10.8%) | 179 (23.3%) | 132 (13.4%) | 227 (16.8%) | 45 (12.5%) | 89 (6.0%) | 170 (4.5%) |
| Dialysis dependence at 90 days | 7671 (83.2%) | 374 (74.9%) | 461 (59.9%) | 770 (78.3%) | 956 (70.8%) | 289 (80.5%) | 1,331 (90.4%) | 3,490 (92.3%) |
| No longer on dialysis at 90 days  (kidney function recovery) | 649 (7.0%) | 71 (14.2%) | 129 (16.8%) | 81 (8.2%) | 168 (12.4%) | 25 (7.0%) | 53 (3.6%) | 122 (3.2%) |

**
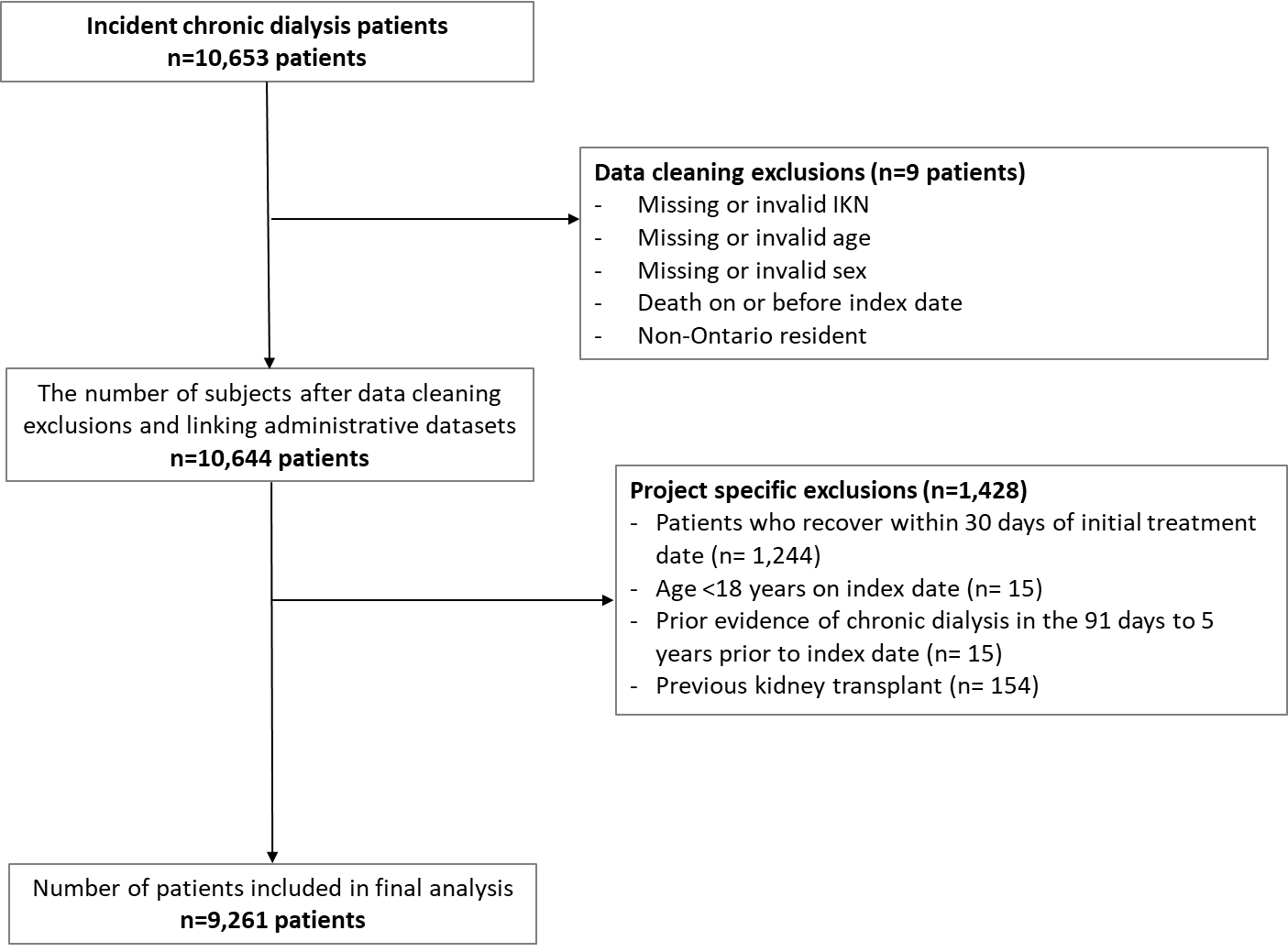
**

**Supplemental Figure 1: Cohort selection**

**Appendix A: RECORD reporting**

# Checklist of recommendations for reporting of observational studies using the REporting of studies Conducted using Observational Routinely-collected health Data (RECORD) Statement

|  | **Item No** | **STROBE items** | **RECORD items** | **Reported** |
| --- | --- | --- | --- | --- |
| **Title and abstract** | 1 | (a) Indicate the study's design with a commonly used term in the title or the abstract.  (b) Provide in the abstract an informative and balanced summary of what was done and what was found. | (1.1) The type of data used should be specified in the title or abstract. When possible, the name of the databases used should be included.  (1.2) If applicable, the geographic region and time frame within which the study took place should be reported in the title or abstract.  (1.3) If linkage between databases was conducted for the study, this should be clearly stated in the title or abstract. | Title and Abstract |
| **Introduction** |  |  |  |  |
| Background/ rationale | 2 | Explain the scientific background and rationale for the investigation being reported. |  | Introduction |
| Objectives | 3 | State specific objectives, including any prespecified hypotheses. |  | Introduction |
| **Methods** |  |  |  |  |
| Study design | 4 | Present key elements of study design early in the paper. |  | Design and Setting |
| Setting | 5 | Describe the setting, locations, and relevant dates, including periods of recruitment, exposure, follow-up, and data collection. |  | Design and Setting / Study Cohort, Indicators and Care Gaps |
| Participants | 6 | (a) Give the eligibility criteria, and the sources and methods of selection of participants. Describe methods of follow-up.  (b) For matched studies, give matching criteria and number of exposed and unexposed. | (6.1) The methods of study population selection (such as codes or algorithms used to identify subjects) should be listed in detail. If this is not possible, an explanation should be provided.  (6.2) Any validation studies of the codes or algorithms used to select the population should be referenced. If validation was conducted for this study and not published elsewhere, detailed methods and results should be provided.  (6.3) If the study involved linkage of databases, consider use of a flow diagram or other graphical display to demonstrate the data linkage process, including the number of individuals with linked data at each stage. | Study Cohort, Indicators and Care Gaps / Appendix D |
| Variables | 7 | Clearly define all outcomes, exposures, predictors, potential confounders, and effect modifiers. Give diagnostic criteria, if applicable. | (7.1) A complete list of codes and algorithms used to classify exposures, outcomes, confounders, and effect modifiers should be provided. If these cannot be reported, an explanation should be provided. | Descriptive Characteristics Prior to Dialysis Initiation by Patient Care Gap Group / Appendix C |
| Data sources/   measurement | 8 | For each variable of interest, give sources of data and details of methods of assessment (measurement). Describe comparability of assessment methods if there is more than one group. |  | Data Sources / Appendix B |
| Bias | 9 | Describe any efforts to address potential sources of bias. |  | N/A |
| Study size | 10 | Explain how the study size was arrived at. |  | Supplemental Figure 1 |
| Quantitative variables | 11 | Explain how quantitative variables were handled in the analyses. If applicable, describe which groupings were chosen and why. |  | N/A |
| Statistical methods | 12 | (a) Describe all statistical methods, including those used to control for confounding.  (b) Describe any methods used to examine subgroups and interactions.  (c) Explain how missing data were addressed.  (d) If applicable, explain how loss to follow-up was addressed.  (e) Describe any sensitivity analyses. |  | Statistical Analysis |
| Data access and cleaning methods |  | N/A | (12.1) Authors should describe the extent to which the investigators had access to the database population used to create the study population.  (12.2) Authors should provide information on the data cleaning methods used in the study. | Author Contributions |
| Linkage |  | N/A | (12.3) State whether the study included person-level, institutional-level, or other data linkage across two or more databases. The methods of linkage and methods of linkage quality evaluation should be provided. | Design and Setting |
| **Results** |  |  |  |  |
| Participants | 13 | (a) Report numbers of individuals at each stage of study--e.g. numbers potentially eligible, examined for eligibility, confirmed eligible, included in the study, completing follow-up, and analyzed.  (b) Give reasons for non-participation at each stage.  (c) Consider use of a flow diagram. | (13.1) Describe in detail the selection of the persons included in the study (i.e., study population selection), including filtering based on data quality, data availability, and linkage. The selection of included persons can be described in the text and/or by means of the study flow diagram. | Supplemental Figure 1 |
| Descriptive data | 14 | (a) Give characteristics of study participants (e.g. demographic, clinical, social) and information on exposures and potential confounders.  (b) Indicate number of participants with missing data for each variable of interest.  (c) Summarize follow-up time (e.g. average and total amount). |  | Results |
| Outcome data | 15 | Report numbers of outcome events or summary measures over time. |  | Results |
| Main results | 16 | (a) Give unadjusted estimates and, if applicable, confounder-adjusted estimates and their precision (e.g. 95% confidence interval). Make clear which confounders were adjusted for and why they were included.  (b) Report category boundaries when continuous variables were categorized.  (c) If relevant, consider translating estimates of relative risk into absolute risk for a meaningful time period. |  | N/A |
| Other analyses | 17 | Report other analyses done (e.g. analyses of subgroups and interactions, and sensitivity analyses). |  | N/A |
| Key results | 18 | Summarize key results with reference to study objectives. |  | Discussion |
| Limitations | 19 | Discuss limitations of the study, taking into account sources of potential bias or imprecision. Discuss both direction and magnitude of any potential bias. | (19.1) Discuss the implications of using data that were not created or collected to answer the specific research question(s). Include discussion of misclassification bias, unmeasured confounding, missing data, and changing eligibility over time, as they pertain to the study being reported. | Discussion |
| Interpretation | 20 | Give a cautious overall interpretation of results considering objectives, limitations, multiplicity of analyses, results from similar studies, and other relevant evidence. |  | Discussion |
| Generalizability | 21 | Discuss the generalizability (external validity) of the study results. |  | Discussion |
| **Other information** | |  |  |  |
| Funding | 22 | Give the source of funding and the role of the funders for the present study and, if applicable, for the original study on which the present article is based. |  | Acknowledgments |
| Accessibility of protocol, raw data, and programming code |  | N/A | (22.1) Authors should provide information on how to access any supplemental information such as the study protocol, raw data, or programming code. | Data Sharing Statement |

**Appendix B: Databases**

| **Database** | **Description** | **Use in Study** |
| --- | --- | --- |
| Canadian Community Health Survey (CCHS) | A national cross-sectional survey that includes information on health status, healthcare utilization, and health determinants. | Descriptive characteristics on smoking history, language and education at the geographic level. |
| Canadian Institute for Health Information’s Discharge Abstract Database/ Same Day Surgery (CIHI-DAD / SDS) | Contains administrative, clinical (diagnosis and procedures/ interventions) and demographic information for all admission to acute care hospitals. | Hospitalizations including procedures and diagnoses for descriptive characteristics and to determine if people are initiating dialysis in the hospital. |
| Canadian Organ Replacement Register (CORR) | Contains clinical and administrative information on individuals with end-stage kidney disease receiving chronic dialysis and people receiving an organ transplant, including information on their donor. | History of kidney transplant (exclusion criteria), and some characteristics including cause of end-stage kidney disease. |
| Congestive Heart Failure (CHF) | Information on all individuals in Ontario with congestive heart failure using a validated algorithm of physician billing claims and inpatient hospital or same day surgery records. | History of CHF for the descriptive characteristics. |
| ICES Physician Database (IPDB) | Information on all physicians who have practiced in Ontario and contains information on demographics, specialty, location of practice and measures of physician activity. | Previous physician visits for the descriptive characteristics and timing of nephrology visits for the indicator and care pathway groups. |
| National Ambulatory Care Reporting System (CIHI-NACRS) | Contains administrative, clinical (diagnosis and procedures) and demographic information for all patient visits made to hospital- and community-based ambulatory care centres (emergency departments, day surgery units, hemodialysis units, and cancer care clinics). | Previous emergency department visits including diagnoses for the descriptive characteristics. |
| Ontario Diabetes Dataset (ODD) | Information on all individuals in Ontario with any type of non-gestational diabetes using a validated algorithm of physician claims and hospital and say day surgery records. | History of diabetes for the descriptive characteristics. |
| Ontario Health Insurance Plan (OHIP) | Contains information on inpatient and outpatient services provided to Ontario residents eligible for the province’s publicly funded health insurance system be fee-for-service health care practitioners and shadow billing for those paid through non-fee-for-service payment plans. | For the cohort creation, to confirm dialysis initiation date and for the descriptive outcome of dialysis dependence at 90 days. |
| Ontario Hypertension Dataset (HYPER) | Information on all Ontario hypertension patients using a validated algorithm of physician billing claims and inpatient hospital or same day surgery records. | Patients with a history of hypertension for the descriptive characteristics. |
| Ontario Laboratories Information System (OLIS) | Laboratory test orders and results from hospitals, community and public health laboratories. | Previous kidney function based on serum creatinine and urine albumin-to-creatinine ratio laboratory values, and other related laboratory tests for the descriptive characteristics. |
| Ontario Marginalization Index (ONMARG) | Geographic-based index developed to quantify the degree of marginalization occurring across Ontario, Canada. It is comprised of four dimensions thought to underlie the construct of marginalization. | Descriptive characteristics on material deprivation. |
| Ontario Renal Reporting System (ORRS) | Contains demographics, clinical and administrative information on individuals with chronic kidney disease and end-stage renal disease. | Cohort of people initiating maintenance dialysis or who received care in a multidisciplinary kidney clinic. Also used to identify dialysis characteristics including modality and location of clinic. |
| Registered Persons Database (RPDB) | Provides basic demographic information for those issued an Ontario health insurance number. The database also provides time periods for which an individual was eligible to receive publicly funded health insurance benefits and the best-known postal code for each resident. | Information on date of birth and sex for descriptive characteristics and date of death for the descriptive outcome at 90 days. |

**Appendix C: Diagnostic and Procedural Codes**

| **Characteristics** | **Databases** | **Codes** |
| --- | --- | --- |
| Chronic dialysis (Exclusion)1, 2 | Canadian Institute for Health Information Discharge Abstract Database (CIHI-DAD)  Ontario Health Insurance Plan Claims Database (OHIP) | Canadian Classification of Health Interventions (CCI): 1PZ21  OHIP fee code: R849, G323, G325, G326, G860, G862, G865, G863, G866, G330, G331, G333, G861, G082, G083, G085, G090, G091, G092, G093, G094, G095, G096, G294, G295, G864, H540, H740 |
| Kidney transplant (Exclusion)3 | CIHI-DAD  OHIP  Canadian Organ Replacement Registry (CORR) | CCI: 1PC85  OHIP fee code: S435, S434  TREATMENT_CODE: 171  TRANSPLANTED_ORGAN_TYPE_CODE [1-3]: 10, 11, 12, 18, 19 |
| Cardiovascular disease (CKD Risk Factor)4-8 | CIHI-DAD  OHIP | International Classification of Disease version 10 (ICD-10): I20, I21, I22, I23, I24, I25, Z955, Z958, Z959, R931, T822, I099, I420, I425, I426, I427, I428, I429, I43, I50, I255, J81, I700, I702, I708, I709, I731, I738, I739, K551, I60, I600, I601, I602, I603, I604, I605, I606, I607, I608, I609, I61, I610, I611, I612, I613, I614, I615, I616, I617, I618, I619, I630, I631, I632, I633, I634, I635, I638, I639, I64, H341  CCI: 1IJ26, 1IJ27, 1IJ50, 1IJ54, 1IJ57, 1IJ76, 1HP53, 1HP55, 1HZ53GRFR, 1HZ53LAFR, 1HZ53SYFR, 1KA76, 1KA50, 1KE76, 1KG50, 1KG57, 1KG76MI, 1KG87, 1IA87LA, 1IB87LA, 1IC87LA, 1ID87LA, 1KA87LA, 1KE57  OHIP fee code: R741, R742, R743, G298, E646, E651, E652, E654, E655, G262, Z434, Z448, R701, R702, Z429, R787, R780, R797, R804, R809, R875, R815, R936, R783, R784, R785, E626, R814, R786, R937, R860, R861, R855, R856, R933, R934, R791, E672, R794, R813, R867, E649  OHIP diagnosis code: 410, 412, 413, 428, 436, 432 |
| Material deprivation (CKD Descriptive Characteristic)9 | Ontario Marginalization Index (ONMARG) | DEPENDENY_CD  Reference data 2016 |
| Diabetes (CKD Risk Factor and Descriptive Characteristic)10, 11 | Ontario Diabetes Dataset (ODD) ICES-derived database | OHIP diagnosis code: Q040, K029, K030, K045, K046  Three OHIP dxcode 250 claims within 1 year |
| Hypertension (CKD Risk Factor and Descriptive Characteristic)12, 13 | Ontario Hypertension Dataset (HYPER) ICES-derived database | ICD-10: I10, I11, I12, I13, I15  OHIP diagnosis code: 401, 402, 403, 404, 405  One hospital admission/same-day surgery (SDS) record with a hypertension diagnosis, or an OHIP claim with a hypertension diagnosis followed by either an OHIP claim or a hospital admission/SDS record with a hypertension dialysis within 2 years. |
| Acute kidney injury (Descriptive Characteristic)2, 14 | CIHI-DAD  OHIP | ICD-10: N17  OHIP fee code: R849, G323, G866, G330, G331, G093, G095, G294, G295 |
| Congestive heart failure (Descriptive Characteristic)15 | Congestive Heart Failure (CHF) ICES-derived database | ICD-10: I500, I501, I509  OHIP fee code: Q050  OHIP diagnosis code: 428  One hospital admission (DAD or OMHRS) with a CHF diagnosis or an OHIP claim/NACRS ED record with a CHF diagnosis followed within one year by a second record with a CHF diagnosis from any source. |
| Dialysis dependence (Outcome)1, 2 | CIHI-DAD  OHIP | CCI: 1PZ21  OHIP fee code: R849, G323, G325, G326, G860, G862, G865, G863, G866, G330, G331, G333, G861, G082, G083, G085, G090, G091, G092, G093, G094, G095, G096, G294, G295, G864, H540, H740 |

Abbreviations: CCI, Canadian Classification of Health Interventions; CHF, Congestive Heart Failure; CIHI-DAD, Canadian Institute for Health Information Discharge Abstract Database; CORR, Canadian Organ Replacement Registry; DEMENTIA, Ontario Dementia Dataset; HYPER, Ontario Hypertension Dataset; ICD-10, International Classification of Disease version 10; ODD, Ontario Diabetes Dataset; OHIP, Ontario Health Insurance Plan Claims Database; ONMARG, Ontario Marginalization Index.

**Appendix D: Indicator definitions**

| **Indicator** | **Criteria used to define the indicator** |
| --- | --- |
| Presence or absence of CKD risk factors | Patients with a first nephrologist visit within <1 year and with unknown nephrology referral eligibility based on insufficient serum creatinine and/or urine ACR measurements in the 1-2 years prior to dialysis initiation were further categorized based on the presence or absence of CKD risk factors (i.e., hypertension, diabetes, cardiovascular disease) (see Appendix B for how these were defined). Ontario recommendations for CKD screening also include first-degree relative(s) with CKD or of Indigenous ancestry, but these factors could not be included due to data unavailability. |
| Nephrology referral eligibility (eligible, ineligible or unknown eligibility) | Patients with a first nephrology visit within <1 year were classified as nephrology referral eligible versus ineligible versus unknown eligibility in the 1 to 2 years prior to dialysis initiation. We used Ontario-specific criteria referred to as the KidneyWise criteria, which include the following:16 two eGFR measurements <30 mL/min/1.73 m2 separated by 90 to 365 days, two random urine ACR measurements >60 mg/mmol separated by 90 to 365 days, an initial eGFR <45 mL/min/1.73 m2 and a second eGFR value in the following 1 to 180 days that showed a decline >5 mL/min/1.73 m2 from the first eGFR value, and a third eGFR in the 14 to 28 days following the second eGFR that was also >5 mL/min/1.73 m2 lower than the first eGFR, or 5-year estimated kidney failure risk17 ≥5% based on an initial eGFR <60, with subsequent eGFR <60 within 90 to 365 days and an ACR either 30 days prior to or 7 days following the second eGFR date. If patients did not have sufficient eGFR and urine ACR measurements, their eligibility was determined to be unknown. |
| First nephrologist visit (inpatient or outpatient) within <1 year versus ≥1 year prior to dialysis initiation. | We looked back 2 years + 90 days prior to dialysis initiation for evidence of a nephrologist visit. |
| Multidisciplinary kidney clinic eligibility (eligible versus ineligible) | Patients with a first nephrology visit within <1 year and who were eligible for nephrology referral were further categorized by multidisciplinary kidney clinic eligible versus ineligible between 1 and 2 years prior to dialysis initiation. As per Ontario guidelines, patients are eligible for multidisciplinary kidney care if they have an eGFR <15 mL/min/1.73m2 or 2-year estimated kidney failure risk ≥10%.18  Patients with a first nephrology visit within ≥1 year were also further categorized as multidisciplinary kidney clinic eligible if they met one of the following criteria: eGFR <15 mL/min/1.73m2 or 2-year estimated kidney failure risk ≥10% between 1 and 2 years prior to dialysis initiation. Otherwise, they were classified as ineligible. |

Abbreviations: ACR, albumin to creatinine ratio; CKD, chronic kidney disease; eGFR, estimated glomerular filtration rate

**References**

1. Clement, FM, James, MT, Chin, R, Klarenbach, SW, Manns, BJ, Quinn, RR, Ravani, P, Tonelli, M, Hemmelgarn, BR: Validation of a case definition to define chronic dialysis using outpatient administrative data. BMC Med Res Methodol*,* 11**:** 25, 2011.

2. Quinn, RR, Laupacis, A, Austin, PC, Hux, JE, Garg, AX, Hemmelgarn, BR, Oliver, MJ: Using administrative datasets to study outcomes in dialysis patients: a validation study. Med Care*,* 48**:** 745-750, 2010.

3. Lam, NN, McArthur, E, Kim, SJ, Knoll, GA: Validation of kidney transplantation using administrative data. Can J Kidney Health Dis*,* 2**:** 20, 2015.

4. Quan, H, Li, B, Saunders, LD, Parsons, GA, Nilsson, CI, Alibhai, A, Ghali, WA: Assessing validity of ICD-9-CM and ICD-10 administrative data in recording clinical conditions in a unique dually coded database. Health Serv Res*,* 43**:** 1424-1441, 2008.

5. Quan, H, Sundararajan, V, Halfon, P, Fong, A, Burnand, B, Luthi, JC, Saunders, LD, Beck, CA, Feasby, TE, Ghali, WA: Coding algorithms for defining comorbidities in ICD-9-CM and ICD-10 administrative data. Med Care*,* 43**:** 1130-1139, 2005.

6. Austin, PC, Daly, PA, Tu, JV: A multicenter study of the coding accuracy of hospital discharge administrative data for patients admitted to cardiac care units in Ontario. Am Heart J*,* 144**:** 290-296, 2002.

7. McCormick, N, Lacaille, D, Bhole, V, Avina-Zubieta, JA: Validity of myocardial infarction diagnoses in administrative databases: a systematic review. PLoS One*,* 9**:** e92286, 2014.

8. Tu, K, Wang, M, Young, J, Green, D, Ivers, NM, Butt, D, Jaakkimainen, L, Kapral, MK: Validity of administrative data for identifying patients who have had a stroke or transient ischemic attack using EMRALD as a reference standard. Can J Cardiol*,* 29**:** 1388-1394, 2013.

9. Tøge, AG, Bell, R: Material deprivation and health: a longitudinal study. BMC Public Health*,* 16**:** 747, 2016.

10. Hux, JE, Ivis, F, Flintoft, V, Bica, A: Diabetes in Ontario: determination of prevalence and incidence using a validated administrative data algorithm. Diabetes Care*,* 25**:** 512-516, 2002.

11. Lipscombe, LL, Hwee, J, Webster, L, Shah, BR, Booth, GL, Tu, K: Identifying diabetes cases from administrative data: a population-based validation study. BMC Health Serv Res*,* 18**:** 316, 2018.

12. Tu, K, Campbell, NR, Chen, ZL, Cauch-Dudek, KJ, McAlister, FA: Accuracy of administrative databases in identifying patients with hypertension. Open Med*,* 1**:** e18-26, 2007.

13. Tu, K, Chen, Z, Lipscombe, LL: Prevalence and incidence of hypertension from 1995 to 2005: a population-based study. Cmaj*,* 178**:** 1429-1435, 2008.

14. Hwang, YJ, Shariff, SZ, Gandhi, S, Wald, R, Clark, E, Fleet, JL, Garg, AX: Validity of the International Classification of Diseases, Tenth Revision code for acute kidney injury in elderly patients at presentation to the emergency department and at hospital admission. BMJ Open*,* 2, 2012.

15. Schultz, SE, Rothwell, DM, Chen, Z, Tu, K: Identifying cases of congestive heart failure from administrative data: a validation study using primary care patient records. Chronic Dis Inj Can*,* 33**:** 160-166, 2013.

16. Brimble, KS, Boll, P, Grill, AK, Molnar, A, Nash, DM, Garg, A, Akbari, A, Blake, PG, Perkins, D: Impact of the KidneyWise toolkit on chronic kidney disease referral practices in Ontario primary care: a prospective evaluation. BMJ Open*,* 10**:** e032838, 2020.

17. Tangri, N, Stevens, LA, Griffith, J, Tighiouart, H, Djurdjev, O, Naimark, D, Levin, A, Levey, AS: A predictive model for progression of chronic kidney disease to kidney failure. Jama*,* 305**:** 1553-1559, 2011.

18. Multi-care Kidney Clinic Best Practices. <https://www.ontariorenalnetwork.ca/sites/renalnetwork/files/assets/MultiCareKidneyClinicBestPractices.pdf>. Accessed August 9, 2022.
